# Getting to Zero: Tuberculosis Elimination in California

**DOI:** 10.1007/s40471-016-0076-6

**Published:** 2016-03-29

**Authors:** Pennan M. Barry, Alexander W. Kay, Jennifer M. Flood, James Watt

**Affiliations:** Division of Communicable Disease Control, California Department of Public Health, 850 Marina Bay Parkway, Richmond, CA 94804 USA

**Keywords:** Tuberculosis, TB elimination, Tuberculosis epidemiology in California, Tuberculosis prevention

## Abstract

This review of tuberculosis epidemiology is intended to provide a historical perspective on the public health approach to tuberculosis (TB) control in California. This historical context offers a lens through which to view current epidemiologic trends and insight into how new therapeutic tools can be applied. Since 1993, the year detailed case reporting was instituted, California has had a decrease in recent TB transmission as evidenced by a reduction in pediatric cases and an increased percentage of cases attributable to progression of latent infection to TB disease in the foreign-born population. Overall, there has been a dramatic decline in the annual TB case count, but the speed of the decline has slowed over the last several years. At the current pace and case count of 2137 in 2015, California will not achieve TB elimination (<1 TB case per one million population) for at least 100 years. There are an estimated 2.1 million persons in California with latent TB infection. Modeling suggests that LTBI detection and treatment are important in reaching TB elimination. For this reason, a coalition of stakeholders in California is exploring novel approaches to accelerate the case decline in order to prevent unnecessary disease and death.

## Introduction

From the gold rush of the 19th century to the Silicon Valley tech boom of the 21st century, the “California Dream,” has drawn millions to the state. Throughout California history, tuberculosis (TB) has been a counterpoint to this dream for a brighter future. Despite remarkable progress against TB over the last 100 years, TB is still diagnosed in California every 4 h. Every other day, a Californian dies with TB, and each week, at least one child under five is found to have TB disease. At a time when tuberculosis is a leading killer among infectious diseases worldwide [1], infecting approximately 1/3 of the world’s population [2], TB paradoxically has become less visible in the USA, competing with waves of newer pathogens that make daily headlines. The fact that TB still causes preventable deaths and disease deserves an invigorated response. This report describes the history and epidemiology of TB in California and presents new strategies that can lead toward the dream of TB elimination (<1 TB case per one million population) in California [1].

## The Pathogen

Tuberculosis is caused by the slow-growing microbe, *Mycobacterium tuberculosis*. The pathogen has achieved its success, in part, because of its ability to lurk for many years in its host before escaping immune system control and multiplying to the point of causing serious harm. Once ill and coughing, the person suffering from tuberculosis disease becomes contagious and the pathogen is spread through droplets released into the air. Just as it was invisible in the body for years, it is transmitted without notice to those who breathe in the droplets coughed out by the ill tuberculosis patient. Once inhaled, the bacteria take up residence in the lungs. However, for up to 90 % of those infected, the bacteria are kept in check by the host’s immune system in a latent stage throughout life. For approximately 5–10 % of these infected hosts with latent TB infection (LTBI), the immune system will not be able to contain the infection and replicating TB bacteria will cause illness (active TB disease).

## History of the Tuberculosis Epidemic in California

In 1910, the population of California was 2.3 million, less than 10 % of what it is today [3]. During this time period, TB caused one in seven deaths and was a more visible killer than cancer and heart disease [4]. Known as consumption, it was familiar to the rich and the poor, the native Californian, and the newcomer. The California Tuberculosis Commission of the State Board of Health described an all too common tragedy in California: “in one family where parents and five children occupied a one-room shack, all but two died of TB in quick succession” [4]. Even before robust public health surveillance, statistics showed TB’s impact in California. A total of 644 TB cases and 453 TB deaths were reported in a single month of 1913 [4]. Measles, scarlet fever, typhoid fever, diphtheria, meningitis, polio, and smallpox all trailed much further behind, with deaths from each of these communicable diseases combined adding to less than one eighth of the number of deaths caused by TB [4].

## Public Health Response

The effect of TB on California communities triggered one of the state’s most effective public health responses. TB disease and death, and its economic impact, shifted from a household conversation to a serious focus among doctors and government agencies. In 1913, the state health commission launched a structured investigation of the TB problem and reported that, “constantly present in this state is [sic] between 40,000 and 50,000 tuberculous patients in active stages” [4]. TB was the leading cause of premature death, and the average age of death from TB was 30. The Commission counted 5000 deaths each year in California and noted that each death terminated only after many months or years of suffering during which the disease could be spread to others [4].

The economic consequences of TB were also noticed. The Commission estimated that the cost of TB to California was over $20 million ($478.8 million in 2015 dollars) each year [4]. Families were devastated and became homeless when the primary wage earner died or could no longer work. Many persons turned to welfare programs and the government invested in the support of Californians with TB and in the hospitals that cared for them.

Medical and public health infrastructure became stronger and concentrated on detecting TB and averting its spread. By 1920, there were well over 100 TB hospitals and sanatoria operating in California. Isolation of TB patients in sanatoria removed many contagious persons from crowded urban and congregate settings. Laws that required doctors to report TB to public health authorities and that compelled isolation helped to interrupt transmission and set the stage for local and state public health programs as well as the modern public health surveillance systems we have today. These public health programs were central in the execution of the state TB commission recommendations.

TB among cattle was also recognized as interconnected to the human TB epidemic. According to the Commission, in 1913, 15–30 % of cows were infected with tuberculosis [4]. Consumption of raw dairy products was thought to be the source of 10–30 % of TB cases in humans [5]. The eradication of bovine TB was initially thought an impossible feat but proved pivotal in driving down TB in humans [5]. Eradication involved federal, state, and local governments and massive testing and culling of cows. Transmission to humans was curtailed by instituting pasteurization. While raw milk was the only milk consumed in 1900, commercially available pasteurized milk became the norm by 1936 [5].

The gains produced by these public health interventions were augmented by development of effective antituberculosis therapy. Especially after the middle of the 20th century when multidrug treatment became available [6], TB disease was driven down further and death became a fate for a minority of those with TB. Treatment shifted from inpatient to outpatient settings. Hospitals, once crowded with TB patients, became sites of care for TB patients with advanced or life-threatening illness. The TB-specific interventions coincided with other advances affecting general health and healthcare which also likely made a difference. For example, improved nutrition may have reduced progression of latent TB infection to TB disease and improved housing and working conditions may have reduced transmission. As a result, from 1913 to 1981, there was a 60 % drop in TB cases and a >90 % drop in the rate of TB in California expressed as cases per 100,000 population.

**Fig. 1:**
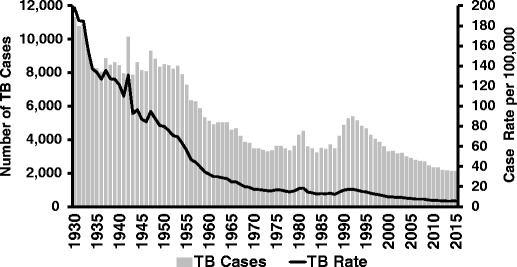
Tuberculosis cases and case rates―California, 1930–2015

However, during the 1980s, the public health infrastructure that was built up previously in the century was neglected [7]. Categorical funding for US TB control programs was halted beginning in 1970. The resource shift away from TB control was followed by a large increase in TB cases in the USA and California (Fig. 1). Several factors contributed to the increase, including a surge in immigration of persons with infection from high burden countries, the vulnerability of HIV-infected persons with weakened immune systems, and relaxing of public health measures and infection control. It became clear that the availability of effective antituberculosis drugs without the ongoing support of public health interventions was not enough to keep TB in check. Once again, government, doctors, and public health programs needed to work together to fight TB.

What ensued was an intensive investment in hospital infection control measures and support for patients to continue TB treatment safely after discharge from the hospital. Investments also included fiscal resources to strengthen local TB programs, expanded coverage of indigent populations with TB by the state’s Medicaid program, and use of new strategies to enable completion of treatment such as housing of homeless patients and directly observed therapy. A more robust TB surveillance system was implemented in 1993 that included reporting of drug susceptibility and TB treatment outcomes. More recently, improvements in immigrant screening have made an additional important dent in case numbers in California [8, 9].

## Current Epidemiology

Today, with approximately 40 million residents, California is the most populous state in the nation and arguably the most diverse [10]. Its large population differs in composition from populations in other states and reflects the migration patterns and countries of origin of migrants over time. Approximately 10 million or 20 % of Californians were born outside the US with Mexico, Philippines, China, and Vietnam being the most common birth countries. California’s foreign born population represents approximately 25 % of all foreign born persons living in the USA, and half of all children in California have at least one foreign born parent [11].

There are more than 2100 TB cases reported in California each year, contributing 20–25 % of the tuberculosis reported annually in the USA. More than 75 % of TB cases in California occur among foreign-born persons. TB cases in California arise through three general mechanisms: importation of active disease from outside the USA, active disease occurring as a result of recent transmission of TB within California, and reactivation of infection acquired long ago. During 2010–2014, 7.5 % of cases occurred among immigrants within 1 year of arrival in the USA. An additional 13 % occurred as a result of recent transmission as defined by the CDC [12]. The remaining nearly 80 % of TB cases in California arose from progression of latent infection to active disease in persons who acquired infection a long time ago and typically far away from California.

## Change in Characteristics of TB Cases in California 1993–2014

Since detailed case reporting was instituted for tuberculosis in 1993, substantial progress has been made in controlling tuberculosis. This progress can be seen in the differences between the number and characteristics of cases reported during 1993–1997 compared with cases reported during 2010–2014. Table 1 shows these differences in demographics, clinical characteristics, and outcomes compared using the Chi-square test. Fewer cases are occurring among children, incarcerated persons, and the homeless. These trends are signs of decreasing transmission of TB in California. A smaller percent of TB cases are infected with HIV, likely the result of improved infection control practices, screening of HIV-positive persons, and most importantly, the advent and widespread use of antiretroviral therapy. The small increase in the proportion of TB cases that occur among healthcare workers is explained by an increase in TB cases among foreign-born healthcare workers. This is likely related to changing healthcare workforce that now includes more foreign-born persons [13].

**Table 1:** Characteristics of reported tuberculosis cases in California during 1993–1997 compared with 2010–2014

	1993–1997		2010–2014		
	*N* = 22973		*N* = 11132		
Characteristic	Number of cases	Percent^e^	Number of cases	Percent^e^	*P* value
Age	42^f^	(28, 60)^g^	51^f^	(33, 67)^g^	<0.0001
Age, foreign-born	42^f^	(28, 62)^g^	54^f^	(37,69)^g^	<0.0001
Age, US-born	41^f^	(27, 58)^g^	39^f^	(20, 59)^g^	0.0040
Children (aged <15 years)	2120	(9.2)	496	(4.5)	<0.0001
Foreign-born	14,992	(65.5)	8707	(78.3)	<0.0001
Site of disease					<0.0001
Pulmonary only	17,324	(75.4)	7605	(68.3)	
Pulmonary + extrapulmonary	1264	(5.5)	1171	(10.5)	
Extrapulmonary only	4382	(19.1)	2355	(21.2)	
Cavitary disease (among pulmonary cases) ^a^	3718	(20.8)	1803	(21.7)	0.1280
Sputum smear + disease (among pulmonary cases)^a^	7392	(45.7)	4476	(54.8)	<0.0001
Not culture-positive (among all cases)	5670	(24.7)	2192	(19.7)	<0.0001
Medical risk factors for TB					
HIV infected	1956	(8.5)	454	(4.1)	<0.0001
Diabetes		^d^	2579	(23.2)	
End-stage renal disease		^d^	410	(3.7)	
TNF-alpha		^d^	93	(0.8)	
Organ Transplant		^d^	83	(0.8)	
Other immunosuppression		^d^	668	(6.0)	
Any medical risk factor (includes HIV)		^d^	3671	(33.0)	
Social risk factors for TB					
Diagnosed in Corrections	943	(4.1)	349	(3.1)	<0.0001
Homeless	1770	(8.4)	588	(5.3)	<0.0001
Drug use	1775	(9.6)	725	(6.6)	<0.0001
Excessive alcohol use	2504	(13.9)	918	(8.4)	<0.0001
Healthcare worker	383	(1.7)	419	(3.8)	<0.0001
US-born healthcare worker	122	(0.5)	70	(0.6)	
Foreign-born healthcare worker	260	(1.1)	349	(3.1)	
Drug resistance^b^					<0.0001
Pansusceptible (includes EMB monoresistance)	14,421	(87.4)	7443	(84.5)	
INH monoresistance	1279	(7.8)	716	(8.1)	
Rifampin monoresistance	101	(0.6)	17	(0.2)	
PZA monoresistance	248	(1.5)	445	(5.1)	
Polydrug resistance	159	(1.0)	66	(0.8)	
MDR	246	(1.5)	103	(1.2)	
Pre-XDR	34	(0.2)	15	(0.2)	
XDR	5	(0.03)	4	(0.1)	
Primary provider of TB care^c^					
Private sector	7094	(32.2)	2361	(35.7)	<0.0001
Public TB clinic	10,853	(49.2)	3650	(55.1)	
Both public TB clinic and private sector	4109	(18.6)	610	(9.2)	
Outcome					
Death before treatment start	674	(2.9)	147	(2.2)	0.0005
Among patients alive at diagnosis and started on treatment^c^					<0.0001
Death after treatment start	1828	(8.3)	481	(7.2)	
Lost/moved/refused/unknown	2222	(10.1)	389	(5.9)	
Moved outside the USA	655	(3.0)	149	(2.2)	
Treatment complete	18,050	(81.7)	5772	(86.9)	

*INH* isoniazid, *RIF* rifampin, *PZA* pyrazinamide, *EMB* ethambutol, *IQR* interquartile range, *MDR* multidrug resistance, defined as resistance to at least INH and RIF, *Pre-XDR* pre-extensively drug resistant, defined as MDR plus resistance to a fluoroquinolone or a second-line injectable medication (amikacin, capreomycin, or kanamycin); polydrug resistance, defined as resistance to at least two first-line drugs, but not MDR

^a^Denominator = pulmonary cases with or without extrapulmonary disease

^b^Mutually exclusive categories among cases tested to at least INH and RIF. Includes cases that acquired resistance on treatment

^c^Denominator = alive at diagnosis and started on treatment. Data in later time period only available for 2010–2012

^d^Only available for 2010 and later

^e^Calculations exclude missing and unknown values from the denominator

^f^ median

^g^interquartile range

There were few meaningful changes in drug susceptibility patterns of *M. tuberculosis* isolates in 1993–1997 compared with 2010–2014. Pansusceptible (susceptible to INH, rifampin (RIF), and pyrazinamide (PZA), if tested) and INH-resistant cases remained stable. However, PZA monoresistance has increased (5.1 vs. 1.5 %). PZA monoresistance is primarily attributed to cases caused by *Mycobacterium bovis*, which has been shown to be increasing in California. TB from *M. bovis* particularly affects Hispanic communities in Southern California and is more common among children [14]. TB from *M. bovis* is likely related to consumption of unpasteurized dairy products made outside the USA. Despite a global rise in multidrug-resistant (MDR) TB cases, the proportion of TB cases in California that are MDR has remained stable at 1–2 % of TB cases. One explanation for this observation is that very few MDR cases are being created as a result of acquired resistance from poor treatment practices in California.

Changes in the age of US-born and foreign-born patients with TB have gone in opposite directions with the median age among foreign-born persons increasing from 42 to 54, whereas the median age of US born TB cases has decreased to 39. These opposite trajectories likely indicate major differences in underlying population risks for TB exposure and progression to active disease. Among foreign-born persons, much TB is occurring among persons with age-related medical comorbidities such as diabetes and end-stage renal disease that increase the risk of progression to active TB disease. TB disease also typically is occurring among long-standing residents of the USA with >75 % of TB cases among foreign-born persons occurring in persons who have been present in the USA for at least 5 years. In contrast, US-born TB patients are more likely to have social or behavioral risk factors for TB such as substance abuse, incarceration, or homelessness. These populations can be more difficult to reach with TB prevention activities and also may be groups in whom TB transmission is concentrated.

Among the most dramatic changes in TB during 1993–2014 has been the fall in TB cases from 5150 cases in 1993 to 2137 cases in 2015 (Fig. 1). During the same period, the annual rate of TB declined from 16.3 to 5.5 per 100,000 population. Sadly, nearly 10 % of persons with TB in California still die and nearly one third of these died before TB treatment could be started.

**Fig. 2:**
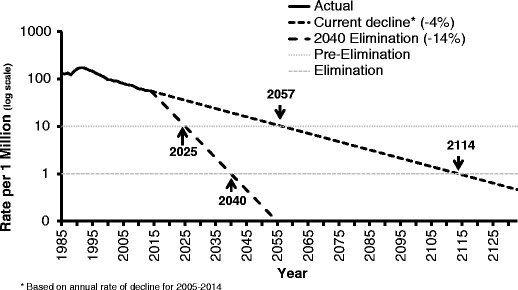
Years of TB pre-elimination and elimination in California. Extrapolation based on current rate of decline

Despite the dramatic decline in TB cases and rates, the speed of the decline has slowed substantially from an average 6.4 % annual decline from 1993–1997 to an annual case decline of 3.8 % during 2005–2014. In the last two years, the decline was only 1 % per year. In 2015, there were 2137 cases of TB reported in California (a rate of 55 cases per million). If the rate of decline from the last 10 years continues, TB elimination (defined as less than 1 case per one million population) will not be achieved for 100 years (Table 2). On the other hand, if the rate of decline can be sped up to 14 % per year, TB could be eliminated from California by 2040 (Fig. 2). Modeling studies have shown that speeding the decline will require addressing the primary source of new TB cases in California, latent TB in incoming immigrants as well as among the population already present in California [15].

**Table 2:** Years to TB elimination and pre-elimination in California

Status	Rate	Cases in California
Current (2015)	55 cases/million	2137
Pre-elimination	<10 cases/million	388^a^
Elimination	<1 case/million	39^a^

^a^Based on 2014 US Census Estimate of California Population: 38.8 million

## Latent TB Infection

In contrast to the epidemiology of TB disease in the USA, the epidemiology of latent TB infection (LTBI) is based on estimates from survey data because LTBI is not a reportable condition. The National Health and Nutrition Examination Survey (NHANES) has been used as a primary source to estimate LTBI prevalence [16]. The NHANES survey has collected data on TB infection through the use of tuberculin skin tests (TST) in the 1971–1972 and 1999–2000 surveys and TST plus interferon gamma release assays (IGRAs) in the 2011–2012 survey. Miramontes et al. analyzed this data set finding that 1.5 and 2.8 % of the US-born population was infected with TB using TST and IGRA, respectively [17••]. However, among foreign-born persons, the prevalence of LTBI was much higher, estimated at 20.5 % by TST and 15.9 % by IGRA. BCG vaccination, common among foreign-born persons, can lead to false positive TST reactions but does not influence IGRA results. That is why IGRA offers superior accuracy in this population.

In order to estimate the number and proportion of California residents with LTBI, prevalence estimates from NHANES 2011–2012 were applied to race/ethnicity and nativity strata derived from California population estimates for 2014 in the US Census Current Population Survey Annual Social and Economic Supplement (CPS; available using the online CPS Table Creator http://www.census.gov/cps/data/cpstablecreator.html). Using TST for US-born and IGRA for foreign-born persons yields an estimate of 2,171,673 persons with LTBI, of which 545,273 are US born and 1,626,400 are foreign born (Table 3). As noted, the use of IGRA more accurately reflects true TB infection in the BCG-vaccinated foreign born population however IGRA may overestimate through false positive results the rate of LTBI in the US-born population with a lower pre-test probability for TB infection [18].

**Table 3:** Estimated prevalence of TB infection in California, 2014^a^

	% with LTBI	2014 population estimate	Number of persons with LTBI
Total	6.1%	38,802,500	2,380,068
US born (all)	1.9 %	27,920,749	542,765
White (non-Hispanic/Latino)	0.7 %	13,933,084	97,532
Black (non-Hispanic/Latino)	5.1 %	2,490,359	127,008
Hispanic/Latino	2.9 %	9,070,625	263,048
Asian	2.4 %	2,086,289	50,071
Other (non-Hispanic/Latino)	1.5 %	340,392	5,106
Foreign-born (all)	16.9 %	10,881,751	1,837,303
White (non-Hispanic/Latino)	9.4 %	1,619,862	152,267
Black (non-Hispanic/Latino)	15.2 %	193,964	29,483
Hispanic/Latino	15.6 %	5,392,958	841,301
Asian	22.3 %	3,592,700	801,172
Other (non-Hispanic/Latino)	15.9 %	82,267	13,080

^a^Based on National Health and Nutrition Examination Survey, 2011–2012, as reported by Miramontes et al. [16] using tuberculin skin test for US born and interferon gamma release assay for foreign born (population estimate from US Census American Community Survey)

Currently, there are several populations that undergo mandatory and systematic assessments for TB infection including approximately 5000 immigrants with abnormal overseas pre-immigration radiographs receiving follow-up evaluations in California and 113,000 foreign-born persons who apply to adjust their immigration status in California to become permanent US residents per year. These groups are at high risk for TB infection and disease because of the likelihood of exposure in their country of origin and warrant systematic screening for TB infection [19]. However, screening and treatment of new arrivals will not fully address the TB problem in California because of the large population of foreign-born persons currently living in California with untreated LTBI. This is in line with recent data from Walter et al. describing the ongoing elevated risk of TB reactivation in immigrants from the Philippines up to 9 years after immigration to California [20••]. Additionally, California TB surveillance data indicates that approximately 70 % of 2014 cases in foreign-born persons occurred in those that immigrated over 10 years prior to diagnosis.

Despite these findings, until now, there has not been a recommendation for systematic screening for LTBI in foreign-born persons living in California. However, many populations at far lower risk of TB acquisition undergo systematic screening. Indeed, more than 80 % of the 1,726,000 individuals undergoing mandatory screening in California each year are healthcare workers who are legally mandated to undergo annual screening for TB infection and disease despite a decrease of TB transmission in hospitals today. The cost-savings and effectiveness of risk-based TB screening rather than universal screening, has been demonstrated in other low risk groups in California including school children and school teachers [21]. Re-focusing resources on screening populations at high risk for LTBI provides an opportunity to further reduce the burden of untreated LTBI in California as a whole.

## Efforts toward TB Elimination

The epidemiology of both active TB and of latent TB infection in California point toward a strategy to achieve TB elimination in California involving increased focus on preventing TB among populations at risk for TB exposure and infection. In May 2015, the California Department of Public Health (CDPH) Tuberculosis Control Branch (TBCB) convened the California TB Elimination Task Force to help craft specific strategies for TB elimination [22]. The Task Force emphasized the need for simple and clear guidance to healthcare providers regarding populations to test for LTBI and treat if infected. The recommendations promote the use of (1) local epidemiologic data to identify and engage populations at risk for TB infection, with a focus on testing of foreign-born residents, immunosuppressed persons at high risk for TB progression, and close contacts to persons with infectious TB [23]; (2) full adoption and implementation of diagnostics such as the interferon gamma release assay (IGRA) to improve test specificity and cost-effectiveness in foreign-born populations [24•]; (3) the use of short-course treatment regimens such as 12 doses of isoniazid and rifapentine or 4 months of daily rifampin to increase rates of LTBI treatment completion [25, 26, 27•]. These measures are designed to speed the decline of TB and decrease the time to TB elimination in California.

Several new opportunities make this a good time to push toward TB elimination: (1) the recently introduced short treatment regimen for LTBI (12 dose regimen of INH/rifapentine) has impressive completion rates [13, 27•] resulting in more TB disease prevented; (2) the interferon gamma release assays, while imperfect, add value and efficiency in detecting latent TB infection in the foreign born since false positives due to BCG do not occur; (3) the expansion of healthcare coverage in California provides access to care for many who previously were not engaged in care and who will benefit from TB prevention; and (4) new national and international understanding of the importance of addressing the LTBI reservoir to advance toward TB elimination [1]. Indeed, a model by Hill et al. suggests that if testing and treating LTBI were increased fourfold, along with reduction of untreated LTBI among new arrivers to the USA, TB cases can be reduced approximately tenfold by 2040 [15].

The ambition to reach elimination in the next 15 years has been stimulated by both evidence and pragmatic considerations. Reaching elimination in California by 2040 will require a 14 % annual decline in TB cases. The steepest decline in a given year in California in the past two decades was 11 %, a value relatively close to 14 %. This value is however more than threefold higher than the recent 5-year average annual decline. How would this change in case decline be feasible? Since little investment has been made to date in systematically testing for and treating LTBI in high risk individuals, an investment now is likely to increase the case decline significantly. In addition, progress has been made globally with declines in TB disease and deaths in many regions [1]. Indeed, the WHO has set ambitious TB elimination goals in their plans for low incidence countries [20••].

Currently, TB disease is at a historic low. Many ask whether it is worthwhile to make an investment in elimination if we expect to eventually get there, albeit in 100 years. For each year in which our case decline does not accelerate, we lose over 200 Californians to TB and spend millions of dollars on direct TB services alone. TB deaths are not inevitable deaths, they are preventable deaths. If we achieve TB elimination in 2040, instead of 2115, we would prevent 4200 deaths and 42,411 cases. Among the millions in California with TB infection, most are not aware they are infected but deserve to know and to have an opportunity to prevent disease from occurring.

## Challenges

In the process of intensifying testing and treatment of latent TB infection, there is some fear that these public health efforts could shift focus away from core TB control activities such as early diagnosis, effective treatment of TB disease, and contact investigation. Continuing these activities while also tackling TB prevention will be a challenge, but one that can be met with cooperation between the healthcare and public health system. Another challenge to TB elimination is that Californians regularly travel to and migrate from world regions with higher rates of TB. However, by working with Federal partners, we may be able to make additional progress. A change in TB screening programs for new immigrants and those adjusting their immigration status to identify LTBI and encourage treatment may result in further case decline. Last, and most important for the world as a whole, is WHO’s elimination plan and serious campaign to achieve zero TB deaths [28]. This indeed is a bold change for a disease that is a top killer in so many countries.

Many questions arise about how to normalize testing and treatment of LTBI in primary care and ensure timely detection and treatment of TB disease when it occurs in the setting of waning experience with TB. The current pressures on the busy clinical practitioner to be everything to everyone while providing a meaningful visit for the person seeking care are daunting. Nevertheless, routinely screening for TB risk and then testing those with risk and ensuring treatment of those infected must occur for TB elimination to become possible. Since the California population is large, new efforts and investments are needed to help make this possible in primary care settings.

## Future Research Directions

Despite recent progress in LTBI testing and treatment, the research to deliver improved tests for infection and really rapid treatment of both LTBI and TB disease stand as the two most critical needs beyond an effective vaccine. Neither IGRA nor TST are highly predictive of progression from LTBI to active disease. Operational research is needed to determine the most effective approaches to screening of LTBI. In parallel, investments globally to reach the millions with disease and infection are paramount.

## Conclusions

California leads in population size and in the number of TB cases reported in the USA. Yet we dream of a state that has abundance of people and diversity, but without a threat of disease such as TB. In 2016, California has an unprecedented opportunity to make progress toward TB elimination. TB disease is at a historic low point and the risk of developing new TB infection in California is now small. The introduction of new shorter treatment regimens for latent TB infection makes preventing TB disease much easier. The trick will be to focus testing and treatment while making it routine. The dream of children living without the terrifying infectious diseases of polio and smallpox has been realized. This is a dream we feel is worth pursuing for tuberculosis.

## References

[1] World Health Organization. Global tuberculosis report 2015. 2015. http://www.health-e.org.za/wp-content/uploads/2015/10/Global-TB-Report-2015-FINAL-2.pdf. Accessed 16 Feb 2016.

[2] Dye C, Scheele S, Dolin P, Pathania V, Raviglione MC (1999). Global burden of tuberculosis: estimated incidence, prevalence, and mortality by country. JAMA.

[3] United States Census Bureau. Resident population and apportionment of the U.S. house of representatives. https://www.census.gov/dmd/www/resapport/states/california.pdf. Accessed 19 Feb 2016.

[4] Report of the California tuberculosis commission of the state board of health. Sacramento: California State Printing Office; 1914.

[5] Olmstead AL, Rhode PW (2004). An impossible undertaking: the eradication of bovine tuberculosis in the United States. J Econ Hist.

[6] Murray JF, Schraufnagel DE, Hopewell PC (2015). Treatment of tuberculosis, a historical perspective. Ann Am Thorac Soc.

[7] Committee on the Elimination of Tuberculosis in the United States, Lawrence G (editor). Ending neglect: the elimination of tuberculosis in the United States. Washington D.C.: Institute of Medicine. 2000.

[8] Lowenthal P, Westenhouse J, Moore M, Posey DL, Watt JP, Flood J (2011). Reduced importation of tuberculosis after the implementation of an enhanced pre-immigration screening protocol. Int J Tuberc Lung Dis.

[9] Liu Y, Painter JA, Posey DL, Cain KP, Weinberg MS, Maloney SA (2012). Estimating the impact of newly arrived foreign-born persons on tuberculosis in the United States. PLoS One.

[10] United States Census. 2010 Census Shows America's Diversity Press Release. 2011. https://www.census.gov/newsroom/releases/archives/2010_census/cb11-cn125.html. Accessed 18 Feb 2016.

[11] Hans J, Mejia MC. Just the facts: immigrants in California. Public Policy Institute of California. 2013. http://www.ppic.org/main/publication_show.asp?i=258. Accessed 12 Jan 2016.

[12] France AM, Grant J, Kammerer JS, Navin TR (2015). A field-validated approach using surveillance and genotyping data to estimate tuberculosis attributable to recent transmission in the United States. Am J Epidemiol.

[13] Lowell BL. The foreign born in the American healthcare workforce: trends in this century’s first decade and immigration policy. Paper prepared for presentation at conference on “Migration and Competitiveness: Japan and the United States” at the University of California at Berkeley, March 2012. 2012. https://migrationfiles.ucdavis.edu/uploads/rs/files/2012/9/ciip/lowell-us-health-care.pdf. Accessed 19 February 2016.

[14] Gallivan M, Shah N, Flood J (2015). Epidemiology of human *Mycobacterium bovis* disease, California, USA, 2003–2011. Emerg Infect Dis.

[15] Hill AN, Becerra J, Castro KG (2012). Modelling tuberculosis trends in the USA. Epidemiol Infect.

[16] Centers for Disease Control and Prevention. NHANES 2011–2012 overview. National Center for Health Statistics. 2014. http://www.cdc.gov/nchs/nhanes/nhanes2011-2012/overview_g.htm. Accessed 10 Jan 2016.

[17.••] Miramontes R, Hill AN, Yelk Woodruff RS, Lambert LA, Navin TR, Castro KG (2015). Tuberculosis infection in the United States: prevalence estimates from the national health and nutrition examination survey, 2011–2012. PLoS One.

[18] Slater ML, Welland G, Pai M, Parsonnet J, Banaei N (2013). Challenges with QuantiFERON-TB Gold assay for large-scale, routine screening of U.S. healthcare workers. Am J Respir Crit Care Med.

[19] Porco TC, Lewis B, Marseille E, Grinsdale J, Flood JM, Royce SE (2006). Cost-effectiveness of tuberculosis evaluation and treatment of newly-arrived immigrants. BMC Public Health.

[20.••] Walter ND, Painter J, Parker M, Lowenthal P, Flood J, Fu Y (2014). Persistent latent tuberculosis reactivation risk in United States immigrants. Am J Respir Crit Care Med.

[21] Flaherman VJ, Porco TC, Marseille E, Royce SE (2007). Cost-effectiveness of alternative strategies for tuberculosis screening before kindergarten entry. Pediatrics.

[22] California Department of Public Health, California Tuberculosis Controllers Association, University of California San Francisco. Report of the California tuberculosis elimination task force meeting, 2015. 2015. http://www.cdph.ca.gov/programs/tb/Documents/TBCB-Report-CA-TB-Elimination-Task-Force-Meeting-2015.pdf. Accessed 19 Feb 2016.

[23] California Department of Public Health, Curry International Tuberculosis Center, California Tuberculosis Controllers Association. California tuberculosis risk assessment. 2015. http://www.cdph.ca.gov/programs/tb/Documents/TBCB-CA-TB-Risk-Assessment-and-Fact-Sheet.pdf. Accessed 12 Feb 2016.

[24.•] Linas BP, Wong AY, Freedberg KA, Horsburgh CR (2011). Priorities for screening and treatment of latent tuberculosis infection in the United States. Am J Respir Crit Care Med.

[25] Villarino ME, Scott NA, Weis SE, Weiner M, Conde MB, Jones B (2015). Treatment for preventing tuberculosis in children and adolescents: a randomized clinical trial of a 3-month, 12-dose regimen of a combination of rifapentine and isoniazid. JAMA Pediatr.

[26] Cruz AT, Starke JR (2014). Safety and completion of a 4-month course of rifampicin for latent tuberculous infection in children. Int J Tuberc Lung Dis.

[27.•] Sterling TR, Villarino ME, Borisov AS, Shang N, Gordin F, Bliven-Sizemore E (2011). Three months of rifapentine and isoniazid for latent tuberculosis infection. N Engl J Med.

[28] Stop TB Partnership. Global plan to end TB: the paradigm shift 2016–2020. 2015. http://www.stoptb.org/assets/documents/global/plan/GlobalPlanToEndTB_TheParadigmShift_2016-2020_StopTBPartnership.pdf. Accessed 19 Feb 2016.

